# Magnesium and diabetes mellitus

**DOI:** 10.12861/jrip.2012.16

**Published:** 2012-09-01

**Authors:** Ali Ghorbani, Azar Baradaran

**Affiliations:** ^1^Department of Nephrology, Golestan Hospital, Ahvaz Jundishapur University of Medical Sciences, Ahvaz, Iran; ^2^Department of Clinical Pathology, Isfahan University of Medical Sciences, Isfahan, Iran

**Keywords:** Magnesium, Diabetes, Lipids

**Implication for health policy/practice/research/medical education:**

Clinical care should focus on increasing dietary magnesium intake or magnesium supplementation to improve metabolic control and prevent dyslipidemia in diabetes individuals.

Diabetes mellitus is a major public health problem thought the world and is growing in populations (1). Previously, we studied the correlation of serum lipids with serum magnesium value in diabetes patients in on 122. In this study we observed, a significant inverse association of serum magnesium level with serum cholesterol and LDL-C (2). Likewise, in the study conducted by Dasgupta *et al*. on one hundred and fifty, diabetic patients, hypomagnesemia was detected in 11.33% of patients. They observed that hypomagnesaemia in diabetes was correlated with poorer glycemic control nephropathy and retinopathy (3). Ahmed Baig *et al*. conducted a study on 60 diabetic patients. They found that the mean serum magnesium value was significantly low in diabetic patients without and with complications when compared with control group. Also, they observed that serum magnesium level in cases with diabetic complications was much lower than those without complications (4). Accordingly, Kocot *et al*. conducted an investigation on 54 diabetic patients. They found, low serum value of magnesium in diabetic subjects in comparison to healthy individuals. They also found, a weak negative association between plasma magnesium and total cholesterol and between serum magnesium and triglycerides in diabetic patients (5). This result attests our previous results (1-4). More recently Mishra *et al*. studied 45 diabetic patients. They found a significant negative correlation of serum magnesium with triglyceride and VLDL-C level and a positive association of magnesium with serum HDL-C too (6). The correlation of hypomagnesemia and insulin resistance in diabetes patients has been documented previously too (7-9). Similarly in the study of 219 diabetic individuals, Rasheed *et al*. observed, serum magnesium had significantly positive association with HDL-cholesterol, while total cholesterol and LDL-cholesterol was negatively associated, albeit non-significantly, with serum magnesium (10). Moreover, an investigation on 550 diabetic patients, showed serum magnesium had significant negative association with glomerular filtration rate (8). Therefore, clinical care should focus on increasing dietary magnesium intake or magnesium supplementation to improve metabolic control and prevent dyslipidemia in diabetes individuals (6-10).

## Authors’ contributions

All authors wrote the manuscript equally.

## Conflict of interests

The author declared no competing interests.

## Ethical considerations

Ethical issues (including plagiarism, data fabrication, double publication) have been completely observed by the author.

## Funding/Support

None.
